# Advancing Genetic Stock Identification of Leatherback Turtles From Foraging Grounds in the Southwest Atlantic: Insights From Nuclear DNA (nDNA) Analysis

**DOI:** 10.1002/ece3.72776

**Published:** 2026-02-23

**Authors:** Laura Prosdocimi, Suzanne E. Roden, Gabriela M. Velez‐Rubio, Alejandro Fallabrino, Milagros López‐Mendilaharsu, Erin L. LaCasella, Peter H. Dutton

**Affiliations:** ^1^ Laboratorio de Ecología, Conservación y Mamíferos Marinos (LECyMM) Museo Argentino de Ciencias Naturales (MACN‐CONICET), CABA Argentina; ^2^ Karumbé Montevideo Uruguay; ^3^ Southwest Fisheries Science Center, National Marine Fisheries Service National Oceanic and Atmospheric Administration La Jolla California USA; ^4^ Facultad de Ciencias, Sección de Oceanografía y Ecología Marina, Instituto de Ecología y Ciencias Ambientales Universidad de la República Montevideo Uruguay

**Keywords:** Atlantic Ocean, conservation genetics, *Dermochelys coriacea*, Genetic Stock Identification (GSI), microsatellites, nuclear markers, population assignment, sea turtles

## Abstract

The leatherback turtle ( *Dermochelys coriacea* ) undertakes extensive migrations between nesting and foraging areas, where it is exposed to threats such as fisheries bycatch, coastal development, and pollution. Although classified globally as Vulnerable by the IUCN, the Southwest Atlantic subpopulation is considered Critically Endangered, with nesting restricted to Brazil. While satellite telemetry and previous mitochondrial DNA (mtDNA) Genetic Mixed Stock Analysis (MSA) studies have indicated that leatherbacks from West African rookeries migrate to foraging grounds off Argentina and Uruguay, the potential for connectivity with rookeries from other regions remains an open question. Genetic Stock Identification (GSI) using 15 nuclear DNA (nDNA) microsatellite markers was conducted on 78 stranded or incidentally caught leatherbacks from feeding grounds off the coasts of Argentina and Uruguay. Assignment analysis results demonstrated that 92% of the foraging leatherbacks originated from Ghana and Gabon in the Southeast Atlantic (SEA), with lesser contributions from the rookeries in the Northwest Atlantic (NEA; 6%) and the Southwest Indian Ocean (SWI; 1%) rookery in South Africa, all with assignment probabilities (AP) exceeding 95%. Our findings corroborate and extend previous mtDNA studies by enhancing the precision of GSI for individuals possessing common haplotypes and by clarifying the unknown origin of individuals with ‘orphan’ mtDNA haplotypes, such as Dc7.1, which were assigned to the SEA rookeries (AP = 99%). Furthermore, we directly assigned one individual, previously of uncertain mtDNA origin (Dc9.1), to the South Africa rookery (AP = 97%), highlighting the need to consider the extension of the SWI Regional Management Unit (RMU) boundaries to Southwest Atlantic waters in future assessments. The absence of detected connectivity with Brazilian nesting populations underscores the necessity for increased sample sizes and the application of advanced molecular markers. These results advance the understanding of population connectivity across oceanic scales and emphasize the crucial role of international collaboration in conservation endeavors.

## Graphical abstract

Genetic Stock Identification (GSI) of 78 stranded or incidentally caught leatherbacks from feeding grounds off the coasts of Argentina and Uruguay was genotyped with 15 microsatellite markers. Assignment analysis results indicated that 92% of leatherbacks in this region originated from Ghana and Gabon, with smaller contributions from the Northwest Atlantic (6%) and Southwest Indian Ocean (1%), all with assignment probabilities > 95%. By improving knowledge of source populations and their links to foraging areas, this study contributes informative data to future refinement of RMU boundaries and establishes a baseline for monitoring fisheries bycatch and other anthropogenic threats, contributing to the protection of critically endangered leatherback populations.

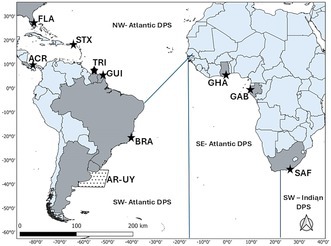

## 1 Introduction

The leatherback turtle *Dermochelys coriacea* , undertakes long‐distance migrations between tropical/subtropical nesting beaches and distant temperate foraging areas (Pritchard 1976; Goff et al. 1994). Leatherbacks occur worldwide, with nesting sites delineated within seven broad subpopulations recognized by the IUCN, which are primarily used for global conservation assessments. Distinct Population Segments (DPS) under the U.S. Endangered Species Act, a legal designation that determines protection status and management at the national level in the United States (National Marine Fisheries Service and U.S. Fish and Wildlife Service 2020). In parallel, Regional Management Units (RMUs) were proposed by Wallace et al. (2010, 2023) to integrate genetic, geographic, and demographic information, providing a biologically meaningful framework for assessing conservation status and threats across ocean basins. All these conservation units are recognized with varying levels of extinction risk. The species faces various anthropogenic threats from fisheries bycatch, coastal development, and marine pollution (Lewison et al. 2004; Saba et al. 2007; Hamann et al. 2010; Wallace et al. 2013; Nelms et al. 2015) resulting in dramatic population declines especially in the Pacific populations (Sarti Martínez et al. 2007; Liew 2011; Tapilatu et al. 2013).

Leatherback nesting is widespread in the Northwest Atlantic (NWA) DPS (Northwest Atlantic Leatherback Working Group 2018; National Marine Fisheries Service and U.S. Fish and Wildlife Service 2020). In contrast, nesting is extremely sparse in the Southwest Atlantic (SWA) DPS, occurring primarily in Espírito Santo, southeastern Brazil, with occasionally nesting in Bahia, Rio de Janeiro, Santa Catarina, and Rio Grande do Sul, and the Parnaíba Delta area in the country's northeastern region (Soto et al. 1997; Marcovaldi and dei Marcovaldi 1999; Barata and Fabiano 2002; Thomé et al. 2007; Loebmann et al. 2008; Tiwari et al. 2013; Bezerra et al. 2014; Colman et al. 2019; Vargas et al. 2019, 2022; Magalhães et al. 2021; Marcovaldi et al. 2021). The Southwest Indian (SWI) DPS likewise contains small nesting assemblages in South Africa and Mozambique (Videira et al. 2011; Nel et al. 2015). While large nesting populations have been documented in Gabon, Ghana, Liberia, and Angola in the Southeast Atlantic (SEA) DPS (Witt et al. 2009; Kouerey Oliwina et al. 2020), these are under pressure from various anthropogenic threats along the West African coast (Witt et al. 2011; Casale et al. 2023).

Satellite telemetry studies have demonstrated that the coastal waters off South America provide important feeding grounds for large juveniles and adult leatherbacks in the SWA (López‐Mendilaharsu et al. 2009; Fossette et al. 2010). This has been documented through frequent records of turtles incidentally captured in artisanal and industrial fisheries, as well as consistent strandings of this species in the area (González‐Carman et al. 2011; Vélez‐Rubio et al. 2013, 2023; Monteiro et al. 2016; López‐Mendilaharsu et al. 2020; Tagliolatto, Goldberg, et al. 2020; Tagliolatto, Giffoni, et al. 2020; Maruyama et al. 2024). For the past 25 years, researchers from Argentina (AR) and Uruguay (UY) have investigated the biology and habitat use of this species in the waters of the Río de la Plata estuary and its maritime front, a coastal feeding area with shared jurisdiction between both countries (López‐Mendilaharsu et al. 2009, 2019; Fossette et al. 2010; Fossette and Witt 2014; Prosdocimi et al. 2016, 2020; Vélez‐Rubio et al. 2023). The estuary of Río de la Plata and its waterfront is a coastal feeding hotspot for the species in the SWA (López‐Mendilaharsu et al. 2009). The abundance of jellyfish prey is believed to support high densities of foraging leatherbacks (López‐Mendilaharsu et al. 2009).

Previous genetic studies using mixed stock analysis (MSA) with mitochondrial DNA (mtDNA) data have shown that leatherback turtles feeding in AR and UY primarily originate from West African breeding populations, particularly the large nesting colonies in Gabon and Ghana (SEA) with mean estimates of 94% (Vélez‐Rubio et al. 2023) and 83% (Prosdocimi et al. 2014), as is also observed in feeding areas off southern Brazil (Vargas et al. 2008, 2019). Stock structure analysis using mtDNA techniques, which characterize maternal lineages within species, has been useful for distinguishing breeding colonies, or Management Units (MUs), across broad geographic scales (Avise 1998). However, these methods may lack the resolution to detect finer scale population structure when haplotype frequency overlap becomes more widespread (Velez‐Zuazo et al. 2008; LeRoux et al. 2012). This is particularly true for leatherbacks, which exhibit low mtDNA variation (Dutton et al. 1999). Dutton et al. (2013) used a panel of 17 nuclear DNA (nDNA) microsatellite loci to distinguish nine demographically independent nesting populations (DIPs) within the seven mtDNA MUs in the Atlantic and SW Indian Ocean. The absence of a clear link between the AR‐UY feeding areas and nearby SWA nesting colonies with previous mtDNA studies is most likely due to the low variability of this genetic marker (Vargas et al. 2022). Satellite tracking studies of post‐nesting females from Espírito Santo, Brazil, demonstrated connectivity between these nesting sites and the coastal and oceanic feeding areas of AR and UY (Almeida et al. 2011; Colman 2018). These findings highlight the need for using additional informative molecular markers to better understand the connectivity of leatherback turtle populations in the SWA. Similarly, telemetry and tagging studies have shown connectivity between the SWI nesting site in South Africa and foraging areas off the African coast in the SEA; however, the connection between this SWI rookery and foraging areas off South America in the SWA is unclear, given the absence of telemetry and tagging evidence and uncertainty of MSA estimates using mtDNA in previous studies (Vargas et al. 2019). This uncertainty is further compounded by the presence of one haplotype (Dc9.1) in three leatherbacks foraging in the SWA that has only been reported in rookeries in the Western and Indo‐Pacific (Toha et al. 2025). This distant connectivity is unlikely, but cannot be ruled out, and there is a need for further investigation in order to clarify the connectivity between rookeries in the Indian Ocean and foraging areas in the Atlantic (see Vargas et al. 2019).

New approaches are now available to assign the population origin of individual turtles using nDNA analysis with possible greater precision than mtDNA MSA (Stewart et al. 2013, 2016; Roden et al. 2017). In this study, we aimed to improve the accuracy of previous MSAs and resolve the uncertainty of mtDNA analysis by using nDNA genotyping (microsatellites) to assign individual turtles to their source nesting assemblage.

## 2 Methods

### 2.1 Study Area

**FIGURE 1:**
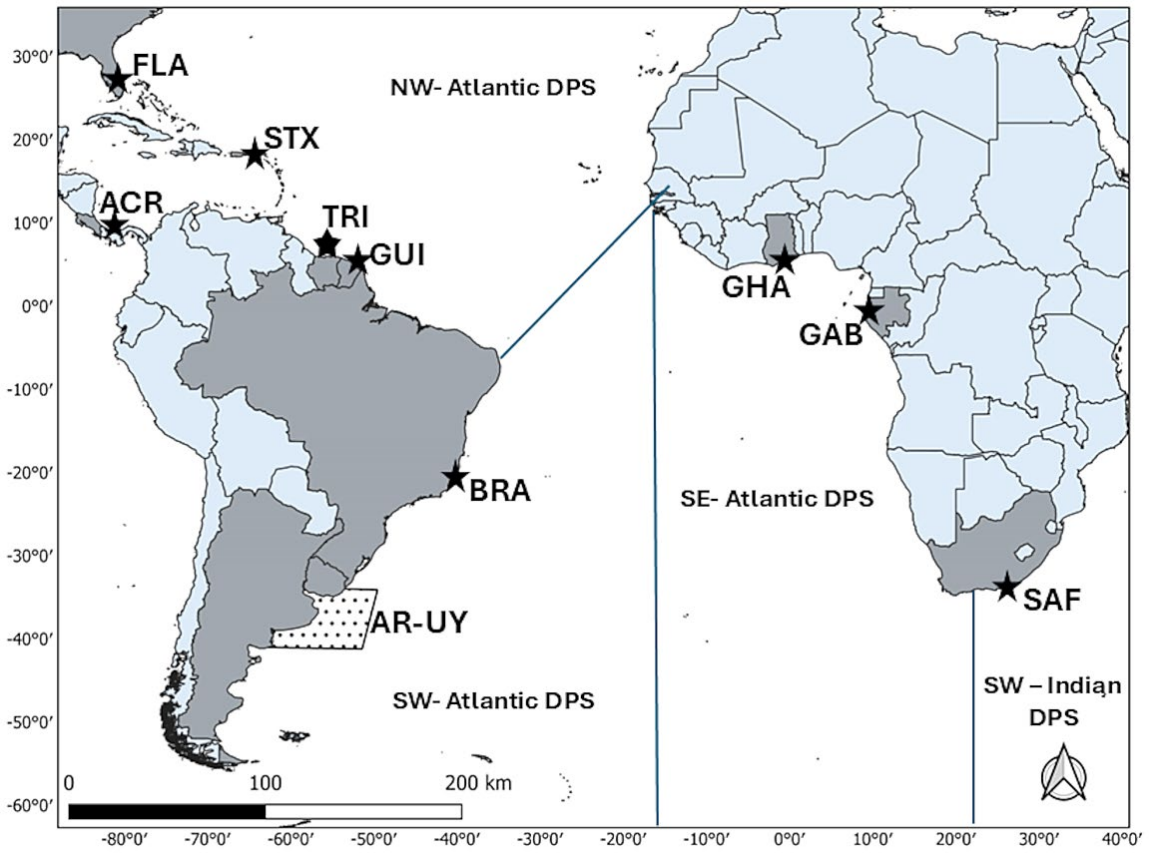
Location of the foraging study area adjacent to Argentina and Uruguay (AR‐UY) indicated by the shaded box. The different DPSes are identified: Southwest Indian (SWI), Northwest Atlantic (NWA), Southwest Atlantic (SWA), and Southeast Atlantic (SEA). Locations of sampled leatherback rookeries (DIPs) within each DPS are indicated by a star, including: Brazil (BRA), Costa Rica (ACR), Florida (FLA), St. Croix (STX), Trinidad and Tobago (TRI), French Guiana and Suriname (GUI), Gabon (GAB), Ghana (GHA), and South Africa (SAF).

The coastal regions of Argentina and Uruguay are part of a complex hydrological system that includes the frontal zone of the Río de la Plata estuary and the Atlantic Ocean (Figure 1). The system is primarily influenced by the confluence of the Malvinas and Brazilian currents and the discharge of the Rio de la Plata estuary (Garcia 1998; Ortega and Martínez Goicoechea 2007), resulting in sea surface temperature variations of more than 15°C (range 10°C–27°C) throughout the year (Acha et al. 2004) and generating a high productivity area (Kruk et al. 2015; Franco et al. 2020). The study area (AR‐UY) includes the waters of the Río de la Plata estuary and the frontal zone including all the coastal waters of Uruguay and part of the coastal waters of the Buenos Aires Province, Argentina (33° 44′ S, 53° 22′ W—36°10′S,58°10′ W).

### 2.2 Genetic Analysis

Tissue samples from stranded or incidentally caught leatherbacks from AR‐UY feeding grounds that were previously characterized with mtDNA (Prosdocimi et al. 2014; Vélez‐Rubio et al. 2023) were reanalyzed with nDNA microsatellite markers.

Standard manufacturer protocols and laboratory procedures were used to extract genomic DNA with one of several extraction methods as described in Dutton et al. (2013) and Vélez‐Rubio et al. (2023). The extracted DNA, in addition to positive and negative controls, was amplified with polymerase chain reaction (PCR) with fifteen polymorphic nuclear microsatellite markers: N32 (Dutton 1995), 14–5, LB110, LB128, LB141, LB142, LB145, LB143, LB133, LB123, LB125, LB157, LB158 (Roden and Dutton 2011) (GenBank: HM217006, HM217009–HM217019); C102, D1 (Dutton and Frey 2009) (GenBank: EU668887–EU668888). Amplification conditions and methods from Roden et al. (2017) were used to genotype the sample set. A genotyping success rate of ≥ 10 out of 15 loci was chosen to prepare a final dataset resulting in 78 individuals for assignment analysis (Data S1). This rate is consistent with previous studies using Dutton et al. (2013) extensive population baseline (Stewart et al. 2013, 2016; Roden et al. 2017) and is more conservative than other cutoffs used in particular with poor‐quality samples (e.g., Morin et al. 2021), due to the high statistical power to detect differentiation of this leatherback turtle microsatellite panel (Dutton et al. 2013).

### 2.3 Data Analysis

A genetic baseline dataset representing nine Demographically Independent Populations (DIPs) of Atlantic leatherbacks (*n* = 1417) (Dutton et al. 2013; Roden et al. 2017) was used to assign foraging leatherback turtles from Uruguay and Argentina (*n* = 78) to probable source DPS using ONCOR software for genetic stock identification (Kalinowski et al. 2007). The four Atlantic and SW Indian Ocean DPS regions included baseline data from the following nine DIPs: Brazil (SWA), Costa Rica (NWA), Florida (NWA), St. Croix (USVI) (NWA), Trinidad and Tobago (NWA), French Guiana and Suriname (NWA), Gabon (SEA), Ghana (SEA), and South Africa (SWI) (Figure 1). Within ONCOR, the baseline dataset described above was used along with the AR‐UY strandings and bycatch as our mixture population. The Individual Assignment option was then used to assign each turtle to a DPS along with its respective calculated probabilities.

## 3 Results

A total of 78 samples were effectively genotyped and used for this study. ONCOR assignment results indicated leatherback turtles foraging in AR‐UY originated from several DPSes in the Western Atlantic, Eastern Atlantic, and Indian Ocean at assignment probabilities > 95% (Data S2). The majority (92%, *n* = 72) of leatherbacks from this study area originated from the West African rookeries of Ghana and Gabon in the SEA. One turtle was assigned to the SWI Ocean region of South Africa. Five turtles (6%) were assigned to the Caribbean Sea region in the NWA. No turtles were assigned to the baseline nesting population in Brazil (SWA).

Of the assigned individuals, 36 had mtDNA haplotypes identified in Prosdocimi et al. (2014) and Vélez‐Rubio et al. (2023). Represented haplotypes included Dc1.1 (*n* = 28), Dc1.3 (*n* = 5), Dc3.1 (*n* = 1), Dc13.1 (*n* = 1), and Dc9.1 (*n* = 1). Turtles assigned to NWA had haplotype Dc1.1 (*n* = 3), to the SEA Dc13.1 (*n* = 1), Dc1.1 (*n* = 25), Dc1.3 (*n* = 5), Dc3.1 (*n* = 1), and to the SWI Dc9.1 (*n* = 1).

Morphometric data were noted at time of sample collection (Prosdocimi et al. 2014; Vélez‐Rubio et al. 2023). Of the leatherbacks successfully genotyped (*n* = 78), 95% were adults, 5% were sub‐adults, and 35% had a biological sex determination; female *n* = 20, male *n* = 7 (Data S2). Within the largest group of assigned individuals (SEA *n* = 72), there were 68 adults and 4 sub‐adults, of which 19 females and 7 males were determined. The NWA group (*n* = 5) were all adults, with one known female. The one individual assigned to SWI was an adult of unknown biological sex (Data S2).

## 4 Discussion

This study builds on previous mixed stock analysis using mtDNA (Prosdocimi et al. 2014; Vélez‐Rubio et al. 2023) by applying assignment testing analysis with a panel of 15 nDNA microsatellite markers with the same sample set. Results of the individual nDNA microsatellite assignments are generally consistent with previous mtDNA MSA, which also show that the majority of the AG‐UY leatherbacks are from West African rookeries (SEA) (Vélez‐Rubio et al. 2023). These results re‐affirm the ability of previous mtDNA MSA approaches to detect strong signals of differentiation when they exist, and conversely validate the utility of the microsatellite GSI approaches to detect that same signal. This is not surprising, given that satellite tracking of nesting females from Gabon also confirms that these nesters migrate to the coasts of Argentina and Uruguay to forage (Witt et al. 2011). However, as Vargas et al. (2019) noted from their study of foraging populations off Brazil, the mtDNA data do not provide sufficient resolution to make detailed inferences about north to south migration from rookeries in the NWA, and this is further reflected in the MSA estimates from the larger mtDNA dataset for the combined SWA foraging sites (Vélez‐Rubio et al. 2023). The median MSA estimated contribution for the NWA rookeries is zero, with 95% Credible Intervals (CI) spanning zero to 10%. This large degree of uncertainty is a characteristic of the mtDNA MSA results for Atlantic leatherbacks, and an inherent limitation of the statistical approach when rookery baselines are dominated by shared common haplotypes (Bolker et al. 2007; Jensen et al. 2013). In contrast, the multilocus nDNA genotype data allowed individual GSI assignment with high confidence, including those with common mtDNA haplotypes present in several DPSes. Taken together with previous mtDNA results, the microsatellite findings advance our understanding of the connectivity between these breeding and foraging areas on opposite sides of the South Atlantic Ocean. Five turtles (6%) assigned to NWA rookeries, confirming connectivity between northern hemisphere breeding populations and southern foraging grounds.

Furthermore, the nDNA analysis allowed GSI assignment of turtles with orphan mtDNA haplotypes previously excluded from MSA. For example, in the previous paper by Vélez‐Rubio et al. (2023), two haplotypes were identified in this sample set (Dc9.1 and Dc1.7) that have not been detected in any Atlantic rookeries to date and were excluded from their analysis. The same samples were genotyped in the present study. The individual with haplotype Dc9.1 genotyped successfully and was assigned with high confidence (97%) to the South Africa baseline rookery (SWI). The sample with haplotype Dc1.7 did not genotype well enough to be included in this analysis. However, we ran an assignment test for that individual with the five microsatellite loci that amplified along with a matching reduced baseline dataset. This resulted in the assignment of the leatherback exclusively to the SEA, West African rookeries of Gabon and Ghana (combined probability > 99%).

It is important to note that, despite the Dutton et al. (2013) leatherback microsatellite baseline including a wide representation of Atlantic rookeries, some rookeries have low sample sizes and others are not yet sampled. This limitation is common across many sea turtle species and highlights the problem of incomplete baselines in assignment studies (e.g., Carreras et al. 2007; Dutton et al. 2013; Shamblin et al. 2014). For Atlantic leatherbacks, the main gaps relevant to our study are in West Africa, particularly rookeries from Equatorial Guinea and Namibia. Orphan haplotypes, such as Dc9.1, also reflect insufficient sampling coverage, as they may be absent from the baseline despite their presence, albeit rare, in the population.

Despite these gaps, assignment results at the DPS level can be interpreted with high confidence. The baseline is sufficiently representative to distinguish broader DPS‐level connectivity, which is highly relevant for management decisions (DPS‐based conservation priorities and RMU frameworks). However, finer scale structure within DPSes or RMUs will require more comprehensive sampling of rookeries to capture rarer haplotypes and improve the precision of rookery‐origin assignments. This is particularly relevant for identifying subtle population structure that may have implications for local management and conservation planning (Vélez‐Rubio et al. 2023).

Finally, it is worth noting that while Dc9.1 has not been reported in any nesting area in the Atlantic or SWI DPS (South Africa) to date, it is common in the western and Indo‐Pacific (Dutton et al. 2007; As‐singkily, Nijland, et al. 2025; As‐singkily, Dutton, et al. 2025; Toha et al. 2025). Our finding that the only reported Dc9.1 foraging turtle assigned to the South Africa rookery suggests that this haplotype is very rare in this SWI DPS and further suggests that the true nesting origin of the two Dc9.1 turtles previously reported from foraging areas near Brazil (Vargas et al. 2019) is the SWI rookeries, rather than the Indo and West Pacific. Further analysis using mixed‐marker approaches is warranted.

The extremely small size of the Brazilian rookery, with an estimated 6–92 nests per year (Thomé et al. 2007), likely requires a much larger foraging ground sample size than used in our study to detect a contribution. Satellite tracking of post‐nesting females in Espírito Santo, Brazil, has recently demonstrated connectivity between these nesting grounds and both coastal and oceanic UR and AR foraging areas and adjoining waters in the SWA (López‐Mendilaharsu et al. 2009; Almeida et al. 2011; Colman 2018). Despite using a more informative molecular marker set, we were still unable to detect this connectivity. While haplotype Dc13.1 has been reported in the nesting female leatherback population in Brazil (Vargas et al. 2022), our study assigned the individual with this haplotype to the SEA composed of the West African rookeries of Ghana and Gabon. The absence of individuals assigned to Brazilian nesting colonies further highlights the extremely reduced size of this population. Furthermore, there is also a small amount of nesting during the boreal summer (May–August) in Northern Brazil, and a satellite track from a single nester that subsequently foraged in the NWA (Magalhães et al. 2021). The seasonal timing of nesting further suggests that the north Brazil nesting might be part of the broader eastern Caribbean nesting (French Guyana) in the NWA. The current genetic baseline for the SWA leatherback rookeries does not reflect the apparent demographic complexity of this small population and needs to be expanded in the future.

The detection of foraging leatherbacks originating from both the NWA and the SWI Oceans, alongside the majority from West African rookeries in the SEA further reinforces the interconnectedness of these populations across vast oceanic scales. These findings highlight the complex connectivity between distant nesting and foraging areas. Satellite tracking studies have shown that leatherback turtles nesting on South African beaches undertake diverse movements influenced by complex oceanographic features, leading them to foraging areas in the South Atlantic Ocean, Southwest Indian Ocean, and Mozambique Channel (Hughes et al. 1998; Luschi et al. 2006; Lambardi et al. 2008; Robinson et al. 2016). Approximately half of these turtles foraged in shallow coastal waters of the Mozambique Channel and northern Madagascar, while the other half moved southward along the Agulhas Current, entering either the Indian Ocean or the South Atlantic. Movements into Atlantic foraging areas off South Africa, Angola, and Namibia overlap with regions used by turtles nesting in Gabon and Brazil. However, genetic evidence indicates no contemporary interbreeding between these populations (Dutton et al. 2013), and tagging data do not show movements between these distant nesting sites.

### 4.1 Conservation Implications

This study provides the first nuclear DNA (nDNA) GSI for leatherbacks in the Southwest Atlantic foraging grounds with a significant finding revealing an individual originating from the Southwest Indian Ocean (SWI). This individual, identified by the mtDNA haplotype Dc9.1, highlights the potential dispersal of SWI turtles across the Atlantic. While this study identified genetic stock origin, these results provide insight into the connectivity between nesting and foraging habitats throughout the Atlantic Ocean. Accordingly, our findings can be of value when assessing and refining RMU boundaries, which, unlike DPSes, may be discontinuous in the marine environment and even overlap in areas where turtles belonging to different rookery RMUs share foraging areas. This significant finding is relevant to future RMU assessments, which should consider re‐drawing the SWI RMU boundary further west, extending into the Southwest Atlantic, including the coasts of Uruguay and Argentina (Wallace et al. 2023).

These results emphasize the importance of integrating genetic and satellite tracking data to refine RMU boundaries and enhance conservation strategies. Satellite tracking in the case of leatherbacks is limited to adult post‐nesting movements of large adults or sub‐adults on foraging grounds, while genetic data, such as those in this study, provide a more comprehensive measure of connectivity shaped not only by adult breeding migrations but also by juvenile dispersal. Understanding the connectivity between distant nesting and foraging areas is critical for effective management of these highly migratory populations.

Ultimately, our results emphasize how threats at feeding grounds, such as fisheries bycatch, can have impacts on nesting colonies located thousands of kilometers away. This underscores the need for international collaboration and regionally tailored management strategies to mitigate threats and enhance the conservation of these migratory species. The genetic approaches used in this study can be further applied to other critical feeding areas for leatherback populations globally, helping to inform threat assessments. Addressing these threats is essential to safeguard the persistence of breeding populations and maintain the ecological role of leatherback turtles in marine ecosystems worldwide.

## Author Contributions

**Laura Prosdocimi:** conceptualization (equal), data curation (equal), formal analysis (equal), funding acquisition (equal), investigation (equal), methodology (equal), project administration (equal), resources (equal), writing – original draft (equal), writing – review and editing (equal). **Suzanne E. Roden:** conceptualization (equal), formal analysis (equal), investigation (equal), methodology (equal), resources (equal), writing – original draft (equal), writing – review and editing (equal). **Gabriela M. Velez‐Rubio:** funding acquisition (equal), resources (equal), writing – review and editing (equal). **Alejandro Fallabrino:** data curation (equal), funding acquisition (equal), resources (equal), writing – review and editing (equal). **Milagros López‐Mendilaharsu:** writing – review and editing (equal). **Erin L. LaCasella:** data curation (equal), writing – review and editing (equal). **Peter H. Dutton:** conceptualization (equal), formal analysis (equal), funding acquisition (equal), investigation (equal), methodology (equal), project administration (equal), resources (equal), supervision (equal), writing – original draft (equal), writing – review and editing (equal).

## Funding

This work was supported by Research supported by: National Marine Fisheries Service (10.13039/100013408); Sistema Nacional de Investigadores‐ Agencia Nacional de Investigación e Innovación; PEDECIBA Biología; National Council for Scientific and Technical Research (CONICET).

## Ethics Statement

This research was conducted under permits (No. 200/04, 073/08, 323/11, 12/14) from the Fauna Department‐MGAP and permits (DF141/16 and 4/2018) from DINAMA‐MVOTMA. This study adhered to the legal requirements of the countries in which the work was carried out and to all institutional guidelines and samples transferred under CITES permits: 002417, 003262, US844694 and US68677C. Genetic analysis was conducted by the Marine Turtle Genetics Program, SWFSC, La Jolla, CA, U.S.A.

## Conflicts of Interest

The authors declare no conflicts of interest.

## Supporting information


**Data S1:** Microsatellite genotype profiles (size in base pairs) for AR‐UY samples analyzed in this study.

**Data S2:** Data S2 ONCOR assignment probability results to DPS regions for AR‐UY microsatellite sample data.

## Data Availability

The data that support the findings of this study are available in the Supporting Information for this article.
